# Composing Well-being: Mental Health and the Mass Observation Project in Twentieth-Century Britain

**DOI:** 10.1093/shm/hkab104

**Published:** 2021-11-06

**Authors:** Andrew Burchell, Mathew Thomson

**Keywords:** mental health, mass observation, autobiography, patient voice, narrative medicine

## Abstract

This article argues that the Mass Observation Project (MOP) at the University of Sussex offers a unique window onto the history of mental health and the voices of those who have lived with mental health conditions during the late-twentieth century. This article analyses how a sample of MOP participants use their writing to reflect on their experiences, and compose narratives about, mental illness over time. More specifically, we suggest that MOP’s capacity for the longitudinal study of individual respondents (underutilised by historians of mental health) offers exciting historiographical and methodological possibilities, not just in the history of mental health but for historians of medicine more generally. We conclude by considering how, for a handful of the participants in the project, mental health is entwined with MOP, as project participants deploy the archive to write about their experiences and even find something akin to therapy in the narrative act.

After years of trying for a family I suffered a depressed pregnancy, followed by 2 years post-natal depression, during which I was also looking after my housebound mother-in-law in her own home (a 6-mile round trip, rarely with car) as she refused to come to live with me. I still have remnants of this bad spell—days of black depression—but now I know it is 48 hours at most & bouts getting rarer. I have, in some part, rebuilt my career & this has helped, but I still get very tense, fast heartbeat etc if I have to write a stiff letter…^1^

Susan (b.1942),^2^ the participant in the Mass Observation Project (MOP) who wrote these words in 1984, typifies the degree of candidness around mental health disclosure found in several members of MOP’s panel of writers. This paper stakes a claim for the MOP Archive at the University of Sussex as a privileged and unique route into approaching the history of mental health in twentieth-century Britain, and particularly with regard to considering the voices and narratives of those who lived, and are living, with mental illness. Through the presentation and analysis of select lives from the archive’s collection, we contend that the value of MOP lies in its ability to offer access to individual voices beyond the institutionalised world of healthcare. We analyse these voices through the ways in which MOP is mobilised as a vehicle for narrating experience and treatment, even being used akin to therapy to find psychological closure. This is especially marked among participants, like Susan, whose involvement with the project has lasted a number of decades and who have thus created narratives that can be read across several directives, ranging from the early 1980s to the late 2010s. While MOP respondents contribute memories and narratives from points in their lives prior to this period, it is not insignificant for any analysis of mental health that the years of MOP’s activity coincide with a major shift in mental health policy in Britain—most especially the apogee of ‘deinstitutionalisation’ and the emergence of more neoliberal modes of mental health intervention characterised by individuation of responsibility for care and treatment.^3^ These years also mark a particular historiographic juncture in the study of mental health in the past through the rise of a more Foucauldian set of interpretations. It is worthwhile noting at the outset that we are keen to avoid our focus on mental health ‘narratives’ and ‘composure’ being mistaken for an ahistorical effort to elide such scholarship, to retrospectively diagnose, to develop a ‘psycho-history’, or to reify individual ‘experience’ of the kind already successfully debunked by Joan W. Scott.^4^ Yet, as scholars of MOP have noted, the very nature of the MOP archive and the intimate, personal narratives contained therein do force its researcher-users to engage introspectively with their own interpretations of human behaviour^5^ in productive ways that can contribute to different currents within the history of psychiatry/history of psy-sciences. MOP’s incidental archive of mental health allows us to explore how individual life experiences and life-course histories shape the narratives and voices of individuals, how such narratives evolve over time, and how they, in turn, allow MOP respondents to recount their experiences. In the process, it asks challenging questions of us as historians about interpretation; how we read such material; and how we respond to the implicit as well as explicit dimensions of mental health, as accessed through textual sources.

Prioritising the voices of those suffering from mental illness was identified as a fruitful area of enquiry for the history of mental health care five years ago, in a collaborative piece by one collective of scholars.^6^ Such work drew from a variety of insights, not least the substantial ‘patient voice’ literature emerging from Roy Porter’s work.^7^ While the field has witnessed engagement with some of the potential routes identified by John Turner, et al. for the patient voice in the late-twentieth-century period, actually accessing the ‘voices’ of psychiatric patients from this moment has only been tentatively explored. The reasons for this are perhaps two-fold. The first concerns the ethical dilemmas of accessing patient records (particularly for research projects with a more contemporary, post-1980 time-frame) and also, perhaps, an apprehensiveness over the appropriateness of oral histories implicating vulnerable populations, especially those likely to disclose information implicating the interviewer in a relationship of trust beyond the normal frameworks of GDPR compliance.^8^ The second, however, reflects the fact that patient voices are, as Turner et al. identified, highly ‘heterogenous’ and almost always refracted through their engagement with the healthcare professionals who frame their responses.^9^ In other words, ‘experience’ is not an independent category. This has been key to the critical engagement with the notion of ‘narrative medicine’ in relation to contemporary mental health, made by Angela Woods and others.^10^ Of particular relevance to some of the MOP respondents discussed below, for example, there has been a profusion of a range of memoirs by survivors of post-natal depression since the late 1990s. All of these occurred with the backing of healthcare professions who contributed commentaries and introductory forwards to the volumes.^11^ The survivor voice here rarely speaks alone and on its own terms, co-opted in the service of medical expertise. Indeed, accessing the voices and feedback of ‘service-users’ has become itself a managerial mantra for mental health provision in the individualising, neoliberal healthcare environment.^12^

It is in this context that MOP—a revival of the older, 1937-*c*.1948 Mass-Observation,^13^ run from the University of Sussex since 1981—is unique as a source-base. In contrast to other initiatives to access the voice of those classified (by others) as patients and ‘service-users’, MOP can be considered an ‘accidental’ or ‘incidental’ repository of writing about mental health—its writing neither seeking to address a ‘specific readership’^14^ of fellow sufferers or professionals, nor its members recruited through externally-imposed medical labelling. Instead, MOP participants are enrolled into the project with the less clear-cut aim of providing an archival repository for ‘everyday life’ in Britain. Life-course narratives can therefore only be constructed in MOP by reading through directives, which lack the public and performative nature of the medicalised ‘recovery narratives’ identified by Woods. Such modes of writing refuse the ‘assumed transparency, neutrality and compulsory positivity’ of directly therapeutic exercises and embrace what Woods and her collaborators term the possibility of more ‘ambiguous’ narratives.^15^

MOP’s panel of correspondents reflect on and write about their own experiences and memories of mental health and psychiatric care in a period from the 1960s to the present through a series of what the project calls ‘directives’. These are open-ended questionnaires on a particular topic, inviting longer, essay-like responses from the panel. Individual respondents are identified, and anonymised, through a personal code, which allows the writing of a particular respondent to be tracked through multiple directive responses. These cover a diverse range of topics and indicate ways in which mental health concerns can be embedded in even quite mundane or unexpected dimensions of everyday life. Through close reading, this allows us to explore how experiences are recounted and narratives of illness and care constructed. Yet, more significantly, the MOP panel also situate themselves in time and space; in longitudinal narratives of selfhood, continuity and change. In this short piece, we suggest that reading the MOP directive responses of a select sample of participants in this way—chronically and synchronically—allows us to explore these two inter-related themes. MOP not only offers a route into exploring individual experiences, but also allows us to understand how memory—as filtered through the unique nature of the MOP archive—grants individual respondents an opportunity to exercise agency in relation to their conditions, control (or otherwise) traumatic memories, and achieve forms of narrative closure which, following the work of the gender and memory scholars Graham Dawson and Penny Summerfield, we term ‘composure’.^16^

Moreover, as we noted earlier, when historians mobilise hospital records—such as nineteenth-century asylum case notes or eighteenth-century memoirs of madness^17^—mental health is refracted through contemporary diagnostic categories and the subjects of our research enter our field of vision as cases that are already classified. Conversely, MOP allows respondents to situate their health in their ‘own’ terms, albeit ones that are heavily influenced by—and thus allow historians to access—the contemporary public languages, labels and scripts of such illness.^18^ In some instances, members of our sample have found some measure of acceptance through these categories. For others, MOP provides a forum through which they can offer resistance to medical diagnoses (or the lack thereof) that are unacceptable to them. Analyses of MOP can thus decentre the institution and the medical professions in the production of patient identity. MOP respondents can be analysed as subjects in their own right; individuals encountering and appropriating medical authority and labels, but without necessarily being filtered, ventriloquised, or framed through them.

## ‘Reading Backwards’: Mass Observation and Method

Using MOP as a resource for ‘memory studies’ is hardly new, but, with the notable exception of Jill Kirby’s work, it is surprisingly under-utilised for the history of psychiatry.^19^ As Tony Kushner notes, the arrival of postmodern theories of memory in the 1990s helped to rehabilitate MOP’s reputation among those who considered its sample of contributors otherwise ‘unrepresentative’.^20^ James Hinton’s two studies of ‘lives’ from both the 1937–*c*.1955 M-O and the MOP of 1981 onwards and, to a lesser extent, Kushner’s attempts to track changing views of migrants and refugees, all suggest ways in which MOP can be read both ‘backwards’ and forwards in time (in the words of Andrea Salter) to elucidate details about life courses, personal trajectories, and thus the wider import of social change over the century as a whole.^21^ More recently, MOP has been mobilised to stake a claim for more complex workings of class and as an adjunct for the history of emotions (although these have yet to take full advantage of the longitudinal possibilities inhering in it).^22^ Work drawing on MOP by Claire Langhamer, Hester Barron and Lucy Noakes, among others—exploring women’s history, wartime experiences, and more general themes of social history through the prisms of memory, generation and gender—have since followed.^23^ Yet, pursuing Hinton’s and Kushner’s more longitudinal analyses of MOP respondents for mental health is unchartered territory for historians. For instance, while Jill Kirby has used both M-O archives to great effect in her cultural history of stress, she does not connect the individual lives of those self-identifying with stress through the archive.^24^

In attempting such an undertaking, we suggest that there are two possible routes, which are not mutually exclusive and are indeed complementary. The first is to amass the range of anecdotal evidence across the directives to (re)construct the life-course of an individual contributor as they seek to present themselves: one which offers a linear progression from birth until (for whatever reason) they cease to write for MOP. Although seemingly ‘linear’, this approach nonetheless recognises that life-courses are shaped by their autobiographical narration; that they are never constantly in movement; and that individual biographies are dynamic and subject to disruption, acceleration and deceleration in given moments—much as Mike Savage and Magne Flemmen’s analysis of social mobility in the National Child Development Study illustrates.^25^ The second potential approach also relies on close reading of directives from particular individuals, but instead explores the specifically *non-linear* dimensions of memory and highlights the agency of the directive-response format itself in the construction of narratives around mental health. To take just one example from our case-studies below: why does a directive on sexuality elicit a more thoughtful and personal response about mental health from one participant than all of the health-related directives combined? With such an approach it is possible to access something that is simultaneously more intimate but also less straightforward to account for: a personal historical consciousness which situates a given respondent in the shifting, lived awareness of their life and health, as well as the competing junctures of historical moments which either offer cultural scripts for making sense of their experiences or else foreclose such narrative possibilities.

Where MOP has been used to explore these issues, the lead has more typically been taken by sociologists, several of whom have been responsible for commissioning the directives which were consulted for our research here. If one of the first to identify the possibilities for medical research inhering in the MOP archive was the medical anthropologist, Robert Dingwall, as early as 1979,^26^ Helen Busby began more longitudinal approaches around the turn of the millennium. Busby mobilised responses to the 1998 ‘Staying Well and Everyday Life’ directive and supplemented these with explorations of individual case-studies from the 1992 ‘Pace of Life’ (commissioned by the psychoanalytically-influenced sociologist, Jenny Shaw) and 1997 ‘Doing a Job’ directives.^27^ What we propose here is something of a similar methodology, albeit on a marginally different scale: following a small, workable selection of individual respondents across a large number of directives. As Salter—another sociologist to make use of M-O—notes, such work requires researchers to use the archive less programmatically and to read it in creative ways that work against the hierarchical organisation of the MOP archive to view constituent contributors in totality.^28^

Ironically, while the archival framing of MOP may be alien to sociologists, these ways of working through MOP—in particular thinking about how we can interpret behaviour to adduce affect and mental state from the material—are unusual for the majority of historians (with the obvious exception of those whose work makes regular recourse to autobiographical material, such as oral historians). Central to our analysis here are two concepts, drawn from cultural and oral history and sociology respectively. The first is Dawson’s and Summerfield’s ‘composure’. We employ this both to reference the material nature of MOP writing as a written ‘composition’ (and one whose genre is difficult to pin down precisely) and to relate individual narratives of mental illness to wider cultural representations and social framing in a deliberately bidirectional, co-produced way—one which allows MOP’s own framework of social production to influence mental health experience. In doing so, we are using the term in a way that remains deliberately agnostic about psychologically-produced knowledge, and therefore our understanding of ‘composure’ is more aligned with Summerfield’s focus on the ways in which autobiography draws from popular tropes (and *vice-versa*) than Dawson’s endeavour to project Kleinian psychoanalysis onto historical actors, or Jenny Shaw’s Freudian reading of MOP participants’ relation to the archive.^29^ The second is a variation on the concept of ‘biographical disruption’ (although, given the longitudinal nature of the MOP archive we prefer the term ‘narrative disruption’) devised by the medical sociologist Michael Bury in the 1980s.^30^ For Bury, writing about diagnostic experience in relation to chronic rheumatoid arthritis, illness constituted ‘that kind of experience where the structures of everyday life and the forms of knowledge which underpin them are disrupted’, and individuals are forced to acknowledge mortality.^31^ The term has been employed by sociologists, exploring ageing and dementia, cancer diagnoses, and other conditions.^32^ However, MOP is capable of adding additional complexity to this analysis by offering an appreciation of narrative shifts over time within the scope of an individual life. In doing so, it accounts for how new cultural scripts—in Summerfield’s sense—and changing discourses around particular symptoms or forms of mental illness can allow participants to minimise the narrative disruption caused by the disease experience.

This approach moves us away from the sole focus on ‘disruption’ identified by Bury, who examined the moment of diagnosis and its immediate sequelae.^33^ As sociologists Chris Sinding and Jennifer Wiernikowski argued over a decade ago, the ‘continuity’ of chronicity narratives, and an ability to rationalise them over time within a life-course, provides one longstanding critique to Bury’s thesis.^34^ Mental health conditions demonstrate this most pertinently but also highlight its complexity, since such conditions affect the very reasoning processes which ‘structure everyday life’ and its rationalisations. Mental illness becomes central to individual biographies but often in highly complex ways, and with degrees of severity that can alter, wax and wane over time. These aspects make it quite different from an arthritis (or for that matter a cancer) diagnosis, even though it is still something for which individuals have to devise strategies for living and around which they develop modes of understanding. As Katherine Foxhall notes of migraine, mental health conditions are ‘medically elusive, noncommunicable disorders … that straddle the boundaries of acute, episodic, and chronic disease’.^35^ Perhaps more so than other conditions, the ‘life stories’ of mental health are therefore ‘layered’ and composed of ‘description and re-description’, as several sociologists working with MOP note.^36^ Once again, these are areas which MOP’s chronological spread over a life-course, encompassing moments of ‘wellness’ as much as ‘illness’, is uniquely poised to address; shifting concern from a disruption of ‘biography’ (as a holistic, empirical concept imposed from without) to the weaving of autobiographical ‘narrative’ itself. If class is, as Mike Savage and Florence Sutcliffe-Braithwaite argue, increasingly a question of narrative and individualised within the framework of a personal trajectory, the same is arguably true for understandings of health and ill-health.^37^ Savage and Flemmen’s suggestion of increasing ‘non-linear’ social mobility, mirrors our own contention that narratives of mental well-being visible in MOP also follow ‘non-linear’ trajectories.^38^

There is a further reason for following particular individuals across several directives: understanding how the context of each directive, as well as the directive format itself, influenced the observer’s production of narrative. According to Dorothy Sheridan, MOP’s former archivist and director, the ‘observer’ role of correspondents inevitably creates tensions—albeit productive ones—within the writings. Observers tend to add a layer of their own interpretation, a kind of ethnographic ‘thick description’, into their own narratives and a ‘dialogue’ between ‘emic’ and ‘etic’ categories is therefore always present.^39^ In short, MOP itself structures responses and can determine whether the respondents write from positions of ‘composure’ or ‘discomposure’.^40^ Busby makes a similar point in relation to the 1998 ‘Staying Well’ directive, emphasising how the respondents constructed narratives that ‘stabilise[d] meanings’ of the self rather than challenged them.^41^ The act of writing for MOP can exert ‘emotional and intellectual demands’ on its authors, forcing them to question received ideas, but it can also cause them to defend a sense of identity.^42^ This aspect of the writing, if read across several directives, offers a window onto the framing of ‘experience’ as a constructed category. Because directives deal with only a single theme, comparing directives reveals how individuals use particular themes to help disclose information about past experiences and to seek composure through the written word. It thus provides a window onto health as well as illness, positive constructions of composure as much as discomposure.

## Constructing the Mental Health Sample

Despite being a methodically organised archive, academic researchers using MOP have long had to endure issues of sampling due to the volume and scope of material that it contains. The size of the panel of respondents has fluctuated considerably over the last few years, although it has progressively decreased.^43^ Random sampling has often been the most frequently employed method, even if the common approach of using surnames beginning with letters ‘A’ and ‘B’ is, as Rose Lindsey and Sarah Bulloch suggest, a somewhat ‘over-used sample’ which risks skewering the types of voices heard through the archive.^44^ Because our initial interest was in experiences of mental health, we decided that the best option was to systematically go through all of the responses to the three directives concerning the NHS (all of which, confusingly, have the same title). We began with the most recent (2018) directive on ‘You and the NHS’^45^ and then worked back to 1997 (also called ‘You and the NHS’) and 2008 (‘You & the NHS in 2008’).^46^ The 2018 directive was commissioned by our project team, ‘The Cultural History of the NHS’, and (as such) contained on overt prompt for respondents about mental health, however we have been unable to go through all of the responses, diminishing its usefulness for this project.^47^

Altogether, there were 299 individual responses^48^ to the 1997 NHS Directive; 248 to its follow-up^49^ in 2008; and 143 in 2018.^50^ In our notes for each directive, we highlighted three different types of MOP contributor. First, cases in which mental health was mentioned directly by the respondent, usually to label their own experiences or those of others (friends or immediate family). Secondly, and often overlapping significantly with the first group for obvious reasons, those who described experiences of mental health services or treatments (once again, for themselves or others). Lastly, there was a nebulous group of individuals whose experiences did not necessarily fit the label of ‘mental health’ precisely but which caught our attention for other reasons pertinent to this research. In this article, the latter group is represented by Lucy (b.1965) who lives with a chronic illness but reflects on her experiences of this through language invoking well-being. The third group was included to suggest ways in which MOP might help to challenge an overly dogmatic and rigidly psychiatry-led approach to defining mental health. In 1997, we identified 27 respondents (or approximately 9 per cent of total responses) who mentioned mental health as per our above caveats. In 2008, despite a decline in the overall number of respondents, 31 (12.5 per cent) mentioned something about mental health and alluded to experience of it. The 2018 directive is more difficult to gauge, both because we included a specific prompt which increases the likelihood of its being mentioned and because—at the time of writing—we have only been able to go through around a third of the directive responses.

Across these Directive replies located at ten-year intervals we developed an initial sample of 54 individuals—35 women and 19 men—to track across other directives. While this may sound an unwieldy number, it should be noted that this figure includes both longstanding and more recent recruits to the project, and that consequently some of those in the sample were found to have contributed very little to the project in general, mental health related or otherwise. This made ‘tracing’ them across directives more straightforward, but generally produced less narrative and biographical depth to the information that we could collect. Male respondents were more likely to be among this group and, consequently, only three men appear in this essay. One way of accounting for this is to consider the gendered nature of MOP itself. Female writers seem to be involved in the project for longer periods whereas male participation is more transitory, thus ensuring that women leave behind a greater depth of life-writing.^51^ Gendered norms may also make men less likely to disclose experiences of mental illness compared to women, while the higher number of women may also be connected to the number of post-natal depression cases within our sample. Perhaps among the first mental health conditions to be discussed more publicly, post-natal depression has become a more popularly resonant narrative to tell and consequently allows writers to attain narrative composure.^52^

Once the sample had been identified, we used the MOP Writer’s Database^53^ to track our sample’s responses to other directives. Although we initially compiled a list of potentially fruitful directives for health and well-being research (with helpful advice and input from the MOP archivists), we decided not to be too dogmatic about sampling all of these. This was partly to circumvent the problem that none of the sample had responded to exactly the same set of directives, but it also ensured that our approach had the flexibility to respond to biographical details and clues within the directives themselves. For example, Patricia (b.1951) alludes to having experienced institutional psychiatric care for anorexia nervosa as a teenager in her responses to both the 2008 and 2018 NHS directives. We consequently cross-referenced this with her 2010 ‘Childhood and Illness’ response, which—as might be expected—offered more autobiographical information about this. (Even if it had not, the silence here might itself have been worthy of analysis as a sign of discomposure within her memories of the event.) More importantly, we felt that there was value—from a purely experimental and historiographical viewpoint—in looking beyond the ‘obvious’ places in MOP where one might expect to locate mental health: that is, outside the directives only concerned with health and ‘well-being’. This was partly to observe how mental health becomes embedded in life experiences and also to highlight the accidental archival and memory spaces where mental health is, to a certain extent, hiding in plain sight. Due to spatial constraints, we cannot provide summaries of all directive questions here—except where they are particularly relevant—but these are available on-line through the MOP webpages.^54^ We would, however, like to draw attention to 2008’s ‘Life Line’ directive (sent out alongside that year’s ‘You & the NHS in 2008’) which specifically, and unusually for a MOP directive, asked for participants to explicitly *narrativise* their life-course, and to represent it either as a list or a diagram.^55^

Our methodology here perhaps offers two responses to the accusation that MOP is somehow ‘unrepresentative’ as a source-base. Although MOP cannot escape entirely from the fact that its panel is weighted towards older, white women,^56^ this is in many respects—and somewhat paradoxically—compensated by its self-selecting nature. A randomly selected sample of questionnaire respondents—even if constructed in the most statistically robust way—would not elicit the same personal reflections that MOP does. The project provides a unique space in which there subsists a relationship between the organisation and its writers and in which the difference between the information that might be collected by MOP and a social survey is consequently measured in kind rather than simply in degree. Moreover, as Dorothy Sheridan notes in relation to an older MOP cohort (that of the 1980s), the question of ‘representativeness’ within the M-O archive is a complex, and possibly moot, one. In the case of the older cohort of female observers, the project may well even constitute a form of historiographical redress, allowing otherwise lost voices (women who considered their educational ambitions to have been ‘de-railed’ by family pressure and institutionalised sexism) the chance to be heard in an accessible historical record.^57^ Indeed, it is tempting to extend Sheridan’s analysis further to include groups such as those in our sample: people with chronic or mental illnesses.^58^

Due to the sensitive nature of some of the material under consideration in the next section, and the fact that reading through several directives provides potentially identifiable biographical descriptions, we have deliberately suppressed or otherwise altered specific geographical details in order to preserve the anonymity of the MOP writers. We have also decided not to identify these MOP participants by their usual codes of numbers and a letter, and instead have assigned pseudonyms which respect stated gender identities. We have provided the MOP archivists with a key, allowing these pseudonyms to be traced back to the MOP code, on the understanding that it will be available for legitimate academic research.

## Self-Narrativizing and Agency in MOP: Defining Mental Health and Recovery

In her study of three directive responses from the 1990s, Busby analysed the role of individual agency in illness through her case studies. Feeling unwell or stressed, she argued, often entails a feeling that agency has been lost, but there were discourses through which respondents could reassert feelings of narrative control over their conditions.^59^ One observer who most exemplifies the potential of MOP to provide narrative and agentic structure is Lucy (b.1965). Her main, self-defined health issues are partial deafness and a diagnosis of myalgic encephalomyelitis (ME)^60^—a controversial condition whose precise form, and even existence, has divided medical expertise and advocacy groups. ME remains central to Lucy’s life throughout her involvement with MOP, visible even through such relatively mundane directives like those on gardening.^61^ One particularly revelatory discussion of her experiences comes in a 2000 enjoinder to ‘Design Your Own Directive’, for which she suggested ‘A View from the Window’: ‘A lot happens outside my window. I’m ill + spend much time gazing out on the world rather than going out + being part of it’.^62^ Her physical symptoms of pain contributed to feelings of depression and suicide, at least at the time of the 1997 NHS directive. In contrast, she appeared to have arrived at a sense of acceptance of her condition in 2008, motivated by the availability of a new thyroid drug which was undergoing trials. Lucy ceased writing for MOP soon after this.

Lucy’s case evidences mental health in the context of other health conditions, and the need to think across medical boundaries. Lucy also experienced her illness as a literal disruption: it emerged after a relatively healthy childhood, and university years spent as a sportswoman. This change in lifestyle clearly exerted an impact on her mental health which came in successive ebbs and flows through the chronological time period of her writing. Her ‘Life Line’ directive response in 2008 reads as a catalogue of her shifting mental state, in which even moments of relative acceptance—in being able to access social housing, for instance—end abruptly with the arrival of a noisy and disrespectful neighbour.^63^ For this observer, ‘being well’ meant effectively managing a condition somewhere on the interstices of mental and physical health, which medical science still cannot adequately conceptualise, and using her own experience as a rhetorical device to overcome prejudices from health-workers and laypeople. Lucy describes how the apparent refusal of medical help is directly experienced; she felt ‘anxious’ to go to her GP surgery in 2008, lest the latest in a changing rota of GPs might ‘know very little about ME’ and perhaps be ‘openly hostile’ to the diagnosis.^64^ Busby’s delineation of agency and capacities for self-control and self-determination were things that Lucy found lacking, although her more belligerent attitude towards medical expertise which denied her lived experience appears to have allowed her an *oppositional* form of agency centred on dissatisfaction with her treatment.

In other cases, respondents found it difficult to attain this kind of composure. For Donna (b.1955), one of several cases of post-natal depression among the sample, giving birth in the 1980s was an experience ‘out of my control’, as doctors and midwives vied with each other to manage the birth and relegated her to a mere passive observer.^65^ Although present from the beginnings of her experience of motherhood, these came more to the fore during the birth of her second son: ‘I felt sick & depressed from the very beginning & had to take time off work because I got so tired’.^66^ In this directive on ‘Birth’, from 1993, her narrative was a still optimistic one of gaining control, but this has diminished, by 1997’s NHS directive, into ‘recurring depression’ over the course of ‘many years’.^67^ Her initial experiences around childbirth had therefore moved towards a more permanent state. It is telling that centring her life around her children was one vital part of attempting a recovery, and may be demonstrated in her decision to submit a directive on ‘children & young people’ to 2000’s solicitation to ‘Design Your Own Directive’.^68^ (Another respondent discussed below, Susan, also found some comfort from this, but over a much longer and historically earlier period of response.)

A perhaps more problematic example of reading MOP responses diachronically for narrative comes in the case of Fiona (b.1957), whose experiences of poor mental health are entwined around her involvement with MOP. Fiona offers a personal perspective on stress and the long-term effects of tranquiliser use, but reading her directive responses backwards raises a set of ethical and procedural questions about how far we can use future admissions of mental illness to read symptoms into earlier responses. In the ‘Pace of Life’ directive (1992), she discloses that nearly a decade earlier she decided to leave her job due to stress.^69^ During her employment, she had begun taking Valium to control this and she remained on the drug throughout the 1980s, an experience she describes as ‘awful’.^70^ A period of unemployment followed, punctuated by occasional employment, the development of agoraphobia, and a career break until 1987.^71^ Around 1990, she became ‘very stressed (for no reason at all that I could see) and went into hospital for 6 weeks to come off tranquilisers’, seemingly on the suggestion of her GP.^72^ Fiona found this a daunting but valuable experience.^73^ During this time, she was seeing a therapist twice a week and by the 1997 NHS directive, she had returned to the same area of employment as when she began writing for MOP.^74^

Since Fiona joined the project in the mid-1980s, do her earlier responses reveal her mental state at this time? In her ‘Views on MO’ (1991)—which, chronologically, would have been despatched to members of the panel broadly conterminously with her hospitalisation—she wrote that she found it more difficult to produce responses about ‘issues such as elections, disasters’ because such topics were ‘not as satisfying as writing about my own experience’.^75^ Fiona suggested that she found something of value in writing for MOP, and it certainly provided a forum—during the 1990s—in which she could work through her past traumatic experiences of stress, agoraphobia and tranquiliser dependency. These were stories that she became adept at telling over and again as she gained narrative composure of her story, and they appear across a variety of different directives throughout the 1990s and early 2000s with marginal changes in emphasis. It is her response to the 1986 directive on HIV and AIDS, however, which reads as a sign of a particularly pessimistic approach at a time in which she was still guarding information about her mental health from MOP. Doubting that medical science would ever locate a cure for the virus, Fiona hoped that she would be ‘old, or dead’ before ‘it will inevitably spread to the rest of the population’. Prompted by the directive to express opinions on contemporary public health education materials about the virus, she felt that such campaigns should foreground more graphic images of ‘emaciated’ patients in the later stages of AIDS and wondered if the virus was simply ‘natures [*sic*] way of cutting the earth’s population for some reason’.^76^ However, the following year’s 1987 response on ‘Holidays’ (at this point she would be about to start a new job) was calmer and more measured, describing attendance at a skills and retraining course for unemployed people.^77^

Should we read Fiona’s response to the 1986 AIDS directive as evidence of a depressive state of mind? This implicates us as historians, and users of the material, in ways of reading that are highly problematic, reading into the form and content of particular writings and diagnosing from a geographical—and temporal—distance. Should a person whose writings come across as rushed, anxious or unstructured be considered as pathological? Perhaps, Fiona’s case is particularly instructive of how MOP offers an opportunity to respond in new ways to the longstanding debates in the history of medicine and psychiatry over retrospective diagnosis. While historians should be sceptical over medical claims to read and impose illnesses into the remote past, as Foxhall notes for migraine, close reading of any historical text does invite consideration of its form, style and content.^78^ Fiona’s later disclosures about her mental health naturally suggest the possibility of such readings, and enable us to understand her later construction of self in opposition to these earlier experiences, within the context of a narrative disruption which denied composure. If we cannot interpret MOP medically with a high degree of accuracy, we nevertheless *can* read it for the absence of this narrative closure, which was certainly true for Fiona: like Donna a decade before, by the time of the 2008 NHS directive, Fiona was afraid that her ‘panics and anxiety are returning’ and was concerned about the waiting lists for counselling and therapy.^79^

Susan (b.1942), invoked at the beginning of this article, is one of the more longstanding and most responsive of our sample, and the respondent who most exemplifies the use of MOP to achieve positive composure (as opposed to the more oppositional form that Lucy demonstrates). When we first came across her in the 2018 NHS directive, her writing mentioned mental health only in the context of the health of others (those in the ambit of her local church). Reading backwards, however, reveals several experiences, ranging from anxiety and nervousness to post-natal depression. In ‘Childhood and Illness’, Susan alludes to a mother who ‘felt let down by a female firstborn’.^80^ She felt that her mother was adept at ‘emotional blackmail’ in order to remove ‘any bid for freedom’ on her daughter’s part,^81^ and that she was therefore ‘always in trouble’.^82^ When Susan experienced a ‘flasher’ after her family relocated across England in the 1950s, she wrote that she could ‘not tell my parents’; partly due to sexual taboo and partly because, due to her ‘mother being of an hysterical inclination, it would also have been my fault it happened in the first place’.^83^ Susan contrasts her own experience with that of her daughter, feeling that whereas the latter had opportunities (partly due to Susan’s reaction against her own upbringing), she was encouraged by her parents to have ‘no spirit or independence’. While Susan was eventually able to leave her family to attend university, she qualified this as ‘Freedom, of a sort’.^84^ A feeling of agency over her life was noticeably absent here, although something had been clearly acquired in later life, judging by the relatively straightforward 2018 directive response which mentioned nothing of her own mental health experiences.

If her description of family troubles and anxiety occupied a later period of Susan’s MOP writing (that of the 2000s), earlier directives from the 1980s and 1990s tended to focus on her experience of post-natal depression. In response to the 1984 ‘Social Well-Being’ directive, she wrote that her ‘depressed pregnancy’ was followed by an awful period of looking after her terminally ill mother-in-law. She referred, in the present tense, to ‘remnants of this bad spell—days of black depression—but now I know it is 48 hours at most & bouts getting rarer’,^85^ a sign that she was beginning to have narrative control over her experiences and could place them into a context of past and present. In 1993’s ‘Birth’ directive she wrote, this time most explicitly in the past tense, that there had been no ‘joys of motherhood’, although she found it difficult to ascribe a cause to her depression. She alluded in this directive to a suicide attempt that was only ‘thwarted’ by the unexpectedly early return of her husband from an appointment.^86^ In Susan’s timeline, however, post-natal depression occurred between and within a longer history of anxiety, panic attacks and migraine, integrating her experiences into a longer life narrative which sought to make sense of her mental health through its physical manifestations. In 1992, writing on her ‘Pace of Life’, Susan referred to feeling ‘tense’ when ‘working against the clock’. She linked having ‘collapsed from sheer tiredness and stress after coping with mother-in-law’ in the early 1980s to her upbringing: ‘I’d made the mistake, very early in life, of not panicking in a crisis and had coped, often unwillingly, with family illnesses since my teens’ with the result that her collapse was retrospectively rationalised as ‘simply saying enough is enough’.^87^ Once again, Susan related this to her parents’ inability to offer her sufficient freedom, which increased her anxiety when she was away from home. This condition began when, as a teenager, she was sent by her parents on her first trip abroad and manifested itself in physical symptoms of terror. Writing about this travel anxiety in the context of the 1987 ‘Holidays’ directive, Susan recalled waking up ‘one night in the hotel there with both legs numb—I was terrified’. The experience left her with recurrent nervous eczema for two years and a debilitating fear of leaving home for overnight travel, which she rationalised in 1987 as a ‘form of agoraphobia’. Susan asserted that the ‘spell’ was finally broken by a particularly active holiday with her husband and friends in which there was so much happening ‘that I never had time to feel worried’.^88^

Susan mobilises a variety of medical languages—from older categories (‘hysteria’), perhaps gleaned during her upbringing, to late-twentieth-century (and still contemporary) categories such as ‘depression’ and ‘anxiety’. These categories perhaps helped her gain composure; to identify, anatomise and rationalise through her writing the symptoms which made her life difficult. Yet she demonstrates, too, how all the respondents explored here often relied/rely on shared cultural scripts—languages which allowed them to explain their experiences to the imagined MOP reader. These languages were surprisingly fluid across different generations, but do betray revealing disparities between gender. Two areas where these were particularly marked lay in discussion of psychoactive drugs (tranquilisers and anti-depressants) and experiences of psychiatric residential care. Such experiences tested the ability to gain a sense of composure, and were most often experienced as an interference with the process of becoming ‘well’.

For example, Susan was lacerating about her doctor’s behaviour during her post-natal depression, which included the prescription of a drug (like Fiona, some kind of tranquiliser), which Susan eventually refused to take because ‘they made me like a zombie’.^89^ Compared to other members of our sample, she was quite fortunate in being able to avoid unpleasant effects with these drugs and her complaints, together with the cultural script of ‘zombification’, are surprisingly common across other members of the sample. Fiona’s main complaint of the tranquilisers echoes this. ‘[C]oming off of those tablets which let you feel nothing was a good thing’, she declared in 1992^90^; while in 1994, she was opposed to the legalisation of soft psychoactive substances due to these experiences.^91^ Describing her experience of this time at the turn of the decade, Fiona foregrounded her own sense of passivity, in which the denial of affect caused by the drugs, far from freeing her from unwanted feelings, was experienced as a denial of her agency:

For 10 years I lived in a dull, grey world, a world of tranquilisers and, at times anti-depressants. Instead of attacking the causes of why I wasn’t coping with my job and then with my life, it was easier to keep me quiet … to take my pain away with drugs … and not by talking as by actions […] I wonder how many people who take soft drugs today (for whatever reason) need talking therapy instead of drugs?^92^

During her tranquiliser use, her addiction was as much to this feeling as the drug itself— ‘[a]nd I always wanted more—more to make me feel number [*sic*]’. Fiona wrote that this feeling, at least initially, gave her a sense of being in ‘control’ when her emotions were perhaps too strong for her but that, by the early 1990s, she was grateful to ‘feel things more now—happiness, pain, hurt, laughter’. ‘Sometimes’, she wrote, ‘that’s good, other times it’s not so good, but I feel I am in control now, not the drugs. Being in control is important to me now’.^93^ Even in her response to ‘Your Life Line’ as late as 2008, she felt that ‘coming off’ tranquilisers marked ‘[o]ne of the turning points of my life’, although it was the only record of her mental health to appear in this autobiographical piece of writing produced eighteen years after her experiences.^94^ In the concurrent response to the NHS directive, she recalled that ‘most of my twenties were spent in a tranquiliser haze’ and described the end of her six-week hospital stay as being ‘on nothing but I was feeling raw and exposed’.^95^

Leaving tranquilisers marked a moment of asserting agency for Fiona, and one which became a powerful memory. Other female respondents mobilised languages of agency, or its absence, in discussing drug use. Trisha (b.1964) felt angry that she had been prescribed unnamed pills (presumably a tranquiliser of some kind) without ‘the full implications of taking the tablets’ being ‘explained to me’, suggesting that one occasion on which she had attempted to give them up in the mid-2000s had resulted in the onset of an immediate depression.^96^ Reflecting gender differences in the experience and articulation of agency, male observers tended to be more secretive about drug use and addiction. Mike (b.1947) was one of the few male observers to write about the experience of feeling addicted to tranquilisers, this colouring his response to the 1994 directive on ‘Drugs’ in which he felt that ‘[m]ental health and happiness are dependent upon a successful encounter with the realities of existence’.^97^ Unlike the female respondents, however, Mike was more keen to extrapolate from his own narrative to offer a cautionary tale for others. He likened opening up about his tranquiliser use to ‘a confession’, a word that he repeated over the course of his (quite short) written response to this directive (and despite the use of medically prescribed tranquilisers hardly being illegal): his tale ‘should reinforce [*sic*] my earlier remarks about the dangers of dependency on mood-altering drugs’.^98^ Whereas women like Fiona, Susan and Trisha saw the prescription of such addictive substances as tantamount to medical negligence which reduced their self-perceived agency and thus led them to discuss their past memories and present experiences in terms of righteous anger, Mike saw it in shameful, emasculating terms. All of them, however, failed to find a sense of composure except through opposition to such treatment.

Other male respondents spoke of a rejection of, and hostility towards taking, medication, which led them to stand their ground with GPs and demand access to counselling. Jon (b.1978) falls into this category and contrasts his experience—the counsellor ‘listened to what I had to say, didn’t rush or belittle me’—with that of his partner, whose GP was not as sympathetic.^99^ Moreover, male observers’ experiences with depression, anxiety and stress were more frequently placed within the framework of work, and they were also less likely to go into details about causes (although reading across directives means that we can piece some of these together). We do not want to suggest that men’s experiences are one-dimensional in this respect. As Ali Haggett and Kirby have noted, simplistic narratives of ‘desperate housewives’ and stressed, working men do not reflect the full diversity of gendered mental health experiences during the twentieth century.^100^ Yet MOP can allow us to trace how men do employ and instrumentalise discourses and narratives of work, often emphasising the work-place disruption over the familial and domestic disruption of mental illness. They also tend to elide discussion of their emotions, in favour of drawing attention to external factors around them. This often led to sharp critique of medical professionals or treatment regimes. Mark (b.1961) was initially diagnosed with depression in 1997 but found his GP evasive and unprofessional.^101^ He found his initial diagnosis through a work scheme which provided private counselling. These colour his experiences and his avowed belief that time-limited intervention approaches to counselling are not sufficiently sustained to be successful.^102^ A member of an older generation, Robert’s (b.1944) depression and stress came from bullying at the hands of a superior at work. Although he was prescribed some kind of medication (he does not specify what), he ultimately felt that it was the ‘human interaction’ with his GP which was more beneficial, particularly because he considered the most serious symptom of his condition was a desire to avoid human contact.^103^ The 1992 ‘Pace of Life’ directive would have been written in the middle of these experiences, and Robert discussed his feeling that he was unable ‘to control outside pressures’, although he placed the blame for this on Thatcherite policy in his area of public-sector employment, which he resented both for political reasons and also because it resulted in an increased workload.^104^ Robert forms a strange example within our sample: a male observer who wrote over a long period, and appears to have gained composure through his writing. This may be connected to his generational position as an older man, from a cohort exposed—even more so than subsequent generations—to psychodynamic theories of mental illness.^105^ His line of employment would also have exposed him to basic training in such theories. As noted, however, male observers tended to reflect far less on mental health in any great detail, and we should be wary of assuming that men’s narratives of illness are homogenous. Indeed, these few voices from the archive are quite diverse in their voice and views, even if they are united in a similarity of (negative) experience.

Institutionalisation offered another area in which observers showed complex relationships to psychodynamic ideas and composure. In contrast to Fiona and Susan, who managed to avoid Britain’s remaining institutions by the time of their breakdowns in the 1980s, Elizabeth (b.1948) was not so fortunate. Elizabeth straddles a generation between Susan (born during the war, and perhaps inheriting an older set of values) and Fiona (who came of age after the marked cultural and social changes of the 1960s and 1970s). Elizabeth’s peers were the most poised to benefit from, and participate in, those changes. Her own left-leaning political consciousness, for instance, is influenced by second-wave feminism, as well as the environmental and anti-nuclear movements.^106^ Her experience of stress came after a marriage break-up in the early 1980s and a work promotion, the latter concomitant with an increase in workload.^107^ Fatigue and insomnia followed, culminating in what she retrospectively identified as a ‘nervous breakdown’ in 1983.^108^ She refers to an unpleasant and traumatic experience of institutionalisation in a psychiatric hospital (a former asylum) in the immediate aftermath of this; her treatment consisting, she claimed, largely of sedative drugs. Elizabeth likened these to ‘liquid cosh’, repeating the same cultural script as Susan and Fiona about how they continued to ‘keep me a zombie for at least two years’ after discharge.^109^ The experience seemingly increased her sense of independence; she became more assertive in refusing ‘to be overloaded at work’ and she states in 1992 that she was able to live ‘in the present with minimal future goals’.^110^ This may well connect to a new-found (and New Age) spiritualism, and she labels herself as a ‘spiritual healer’ in her biographical summary at the start of the 1997 NHS directive. Surprisingly, however, Elizabeth hardly ever explicitly brings her feminist consciousness into her MOP discussions of mental health, from which we can infer either a separation of experience or a lack of composure. Echoing Barbara Taylor’s recent argument, Elizabeth felt that she was ‘lucky’ to have been able to access individual psychotherapy and residential care. She also wrote that it was ‘corrupt & criminal’ to close down and sell the land of the old psychiatric hospitals and asyla.^111^ Here, like Taylor’s discussion of Friern, is a voice which misses the security and calm of the asylum setting, even though her own memories liken her treatment to incarceration and receiving something as physically unpleasant and violating as a ‘cosh’.^112^

Another voice with experience of the psychiatric hospital was Patricia (b.1951), who suffered from anorexia nervosa in her mid-teenage years and was hospitalised in 1966.^113^ Patricia’s most intimate and emotive description of this experience comes in 2010’s DR 90 (‘Childhood and Illness’). She references a feeling that her ‘body … seemed to be “out of control”’ coupled with an awareness ‘of many tensions and conflicts in my life’. At first, in fact, she felt that the condition was simply ‘difficulties in adjusting to adult life’.^114^ From the perspective of later life she appreciates that the condition was sufficiently ‘unheard of’ at the time of her teenage years to make obtaining a diagnosis difficult, with one medical specialist described as ‘gruff’ and insensitive, telling her to ‘“go home and get a good meal down you”’. The resulting family tensions and a lack of resolution resulted in admission to an adolescent unit within a psychiatric hospital, a place where the staff (a nursing sister who ‘ruled the ward’) and patients were mixed. She referenced ‘the trauma’ of:

being in that rambling Victorian psychiatric hospital with its locked ward and its strange men and women wandering in the grounds, one woman walking two steps forward and one backwards, another searching for fag ends and a man walking with an odd gait talking to himself.^115^

Her memories relied on a similar cultural script of the asylum which foregrounded its architectural (but also, by implication, emotional) ‘Victorianism’. Yet, Patricia’s experiences also reveal the decidedly non-Victorian Freudianism of her psychiatrist, who made her life more difficult by relating her eating disorder to an unresolved relationship with her father^116^ and attempted to force her to admit to masturbation.^117^ In her response to 2015’s ‘Dear 16 Year Old Me’, she was able to offer her own interpretation of the condition through her later turn to Buddhist philosophy: a ‘terrible anguish’ resulting from ‘the fact that you have always been supremely sensitive to the subtleties of life and flows of energy that run between people’. Unlike the Freudian, who linked her symptoms to a father-figure, she instead connected them to her mother; having been ‘absorbing your mother’s grief and anger, which she had no way of expressing but of which you were so acutely aware’.^118^ For Patricia, the ‘asylum’ and hospital were ambivalent, if not outright negative, spaces of care and her descriptions of them mobilised a culturally familiar image of the Victorian carceral asylum and the violence associated with it. Patricia’s preferred therapeutic technology, like Elizabeth’s, was esoteric philosophy and religion and she was particularly scathing of Freud’s overdetermination of male sexuality in a way that might make Deleuze and Guattari proud. Nonetheless, both women, like Robert, were part of a generation raised during the zenith of psychodynamic approaches in Britain and it is telling even their turn to religion mirrors a desire for introspection and self-analysis.

Observers who had multiple experiences of residential care tended to be more cognisant of changes over time, usually hinting at improvements. Barbara (b.1948) referred to two experiences of institutionalisation in 1969 and 1982, noting changes in the hospital structure each time. In the course of her response to the 1993 ‘Drugs’ directive she describes her 1970s experience as being characterised by ‘heavy medication and Electro-Convulsive Therapy’ for two months of hospital stay, followed by three months in a ‘rehabilitation unit’.^119^ Like Fiona, she was particularly condemning of the regime of the ‘anti-psychotic drug’, continuing addiction to which she blames for her subsequent inability to start a family.^120^ She was more positive about her second experience of hospital treatment for mental illness in her response to the 1997 NHS directive, and also offered more information on her earlier experience; indicative, perhaps, of achieving some kind of narrative closure on the experience.

What emerges most forcefully in these recollections of residential care experiences is a sense of how the discourses mobilised against the ‘asylum’—though in reality, given the time-frame of the responses, ‘psychiatric hospitals’—entered popular consciousness during the mid-century, providing a set of narrative assumptions and cultural scripts to describe experiences. A few observers (like Barbara) were able to comment on changes across time and reflect more positively on later experiences of residential care, even considering it part of a cure. These positive experiences may have contributed to a sense of narrative composure. By contrast, observers whose experiences (like Patricia’s) were confined to singular periods of incarceration tended to reflect back on these moments more negatively, relying on the same longstanding tropes to narrate their stories. In all of the examples cited, agency over treatment was considered important. This is undoubtedly a consequence both of a late-twentieth-century, neoliberal turn towards placing more responsibility on the individual for healthcare, in tandem with what Busby identified as pervasive feelings of ‘guilt’ about the disruptive potential of illness (particularly in the workplace) in responses from the 1990s, as well as the rise of patient advocacy alongside this.^121^ Asserting control over one’s health, as in Fiona’s case with her emotions under tranquiliser use, became a dominant narrative, but one that appears to have been gendered. It is telling that the men in our sample offered less detail about their health overall, and less insight into how they understood its causes. This may be because they saw mental illness as something external (imposed by work-related stress) rather than internal (coming from pressures of family and upbringing). Such differences in experience also reflect differences in composure: male experiences of mental illness, perhaps conform more towards Bury’s model of total biographical ‘disruption’—due to its ability to affect a masculine sense of self—as opposed to disruptions to ‘narrative’ coherency that are easier to work through. This is particularly marked when we consider the value of MOP to its participants as a therapeutic tool: all of the examples cited in the next section come from female respondents.

## MOP as Therapy: The Agency of the Directive Response

If one conclusion emerges from the scholarship surrounding MOP—and particularly the work undertaken by Sheridan, Brian Street and David Bloome—it is that the project and its format are not transparent recordings of the views and experiences of participants but implicated within the production of knowledge.^122^ How correspondents negotiate their writing and comprehend the logic underpinning it, whether they interpret it as a form of confessional culture or as an historical document, are integral to MOP and worthy of scholarly attention. To these we would add that MOP can be explored as much as an archive of therapy as an archive of affect; that by addressing an invisible audience, participants are able to construct narratives that allow them to create a kind of composure (as a historical subject and as an individual living in a present-moment).

Thinking of MOP as a form of therapy may not be as abstract as it initially appears. The original M-O (1937–1955) was understood by its founders as what Nick Hubble terms a revolutionary form of ‘social therapy’ whose very act of critical observation would have the capacity to radically alter the lives both of its participants and the society around them.^123^ More recently, sociologists such as Hubble and Tew and Susan Benbow and Paul Kingston—as well as the ‘think-tank’ Demos—have explored the use of narrativization and ‘narrative production’ as a tool for older people and the families of those with dementia to reflect on their treatment, offer feedback to their carers, and perhaps derive some relief from doing so.^124^ Hubble and Tew’s work derives in part from engagement with MOP, as well as other literacy-based and life-narrative initiatives with older people. As historians, we are less interested in (nor qualified to comment upon) the viability of MOP as a therapeutic method for treatment. Instead, we seek to identify examples where contributing to the archive appears to have provided help and assistance to particular respondents, through the way in which it allows the creation of narrative, as a way of advancing the possibilities inhering in the historical analysis of mental health and illness within the archive.

Susan provides the clearest and most explicit case within our sample of how a self-narrative of recovery not only occurred in parallel with MOP but also is actively mobilised around it. As we noted earlier, Susan began writing for MOP at the time of her post-natal depression, submitting material from the first directive in 1981. For Susan, whose pre-marriage career had involved writing, contributing this initial piece was—she wrote in response to a 1990 directive soliciting opinions on MOP itself—a test of whether or not ‘I could still concentrate enough to string words together’. She wrote that it ‘gave me confidence’ and eventually persuaded her to return to work once childcare provision allowed.^125^ Seventeen years later, asked to provide a ‘life-line’, this memory seemed still raw for Susan. She stated that her involvement with the project began when she saw a newspaper advert for MOP participants and resolved to contribute something ‘as post-natal depression has sapped my will to live and I want to know if my old skills [i.e. writing] are still there’, before noting that a ‘warm response from Prof David Pocock [then director of MOP] is very encouraging’.^126^ (This was not unusual, Pocock had a policy of replying to all new MOP recruits personally.^127^) It is revealing here that she narrativises this part of her life in the present tense and is one of only a handful of observers in our sample to actually include participation in MOP on their timelines for DR 82 ‘Your Life-Line’, indicative of its importance to her. Contrasting the value of writing for MOP with her GP’s advice to take tranquilisers, Susan ascribed her own recovery from depression to the fact that ‘I made myself begin to write again […] I think forcing my brain into action was better than any drugs’.^128^ Given the role of MOP in her recovery, it is unsurprising that Susan stated a preference for the more personal and probing directives because ‘I feel they are more in the spirit of mass observation’ and acknowledges her MOP writing as a place to hone uncomfortable self-truths.^129^ While this is not a pleasant experience for Susan, she ultimately feels that the emotional honesty of the ‘mass observation’ genre of writing is more important than creating a more palatable version of the self: ‘to set down other than what I felt would have been dishonest’.^130^

Perhaps most revelatory in Susan’s case is the existence of a dual chronology. There is, on the one hand, an historical narrative of recovery from post-natal depression and anxiety occurring in a broadly linear, though not always constant, sequence. There is equally, however, a narrative of memory; of *when* it becomes possible to describe remembered experiences and divulge details to the imagined MOP reader. Such work corroborates longstanding scholarship—such as Andrea Salter’s multitemporal readings of MOP—on the ‘movements back and forth within and across time’ that are visible both in the framing of individual pieces of writing by project participants and around the time-based constraints of the archive itself (the date of a particular directive, versus the life-course of the individual respondent, overlaid with wider historical events and processes).^131^ For Susan, this occurred quite early on in her writing for MOP and appears to be relatively straightforward: the 1987 ‘Holidays’ directive for her anxiety, and the 1984 ‘Social Well-Being’ directive for her post-natal depression. This is not to suggest, of course, that the narrative of memory is always linear, nor, as Susan demonstrates with her separate experiences of travel anxiety and depression, is it always applicable to the same subjects within the same time-frame.

Moreover, at the same time that Susan is quite open about her mental health experiences, she remains more guarded in other details of her private life. Reading her responses to the 2005 directive on ‘Sex’, it is clear that she experienced discomfort in writing, preferring to implicate herself less as an agent and instead to use her moral and religious worldview to frame a response about the behaviour of others. Indeed, she states that she was initially hesitant to answer the directive at all.^132^ Fiona’s case is similar. While she obtained some degree of composure from discussing her experiences of tranquiliser use and the value of talking therapy to help her overcome her childhood, she gives little further information about the youthful experiences that she blames for her condition and they remain invisible in other parts of her MOP writing. Fiona’s ‘Birth’ directive likewise details the trauma of a succession of miscarriages following her weaning from Valium, which are never mentioned again.^133^ By paying attention to how the capacity to *engage* with memory, as well as the remembered *content* of memories themselves, oscillates between directives, it is possible not only to gauge a broader chronology of health and pathology (physical or psychological), but to understand how certain stories become more easy and less emotionally charged to tell over time (or otherwise).

In doing so, questions of retrospective diagnosis are re-imagined through MOP: it is no longer a case of reading depression or anxiety into the text—since these are labels that the individual in question openly embraces—but of reading for composure and the subtleties of narrative. This implicates the historian, but in ways that are productive, linking the processes of reading to the cultural context of writing. In the case of Lucy, who began to write for MOP at a time when her ME was particularly severe and her own mental health suffered as a consequence, it is clear (as we noted earlier) that she experienced something cathartic in being able to recount her experiences of medical care and professional indifference or hostility. As with Susan, Lucy’s strength of voice, once discovered, continued throughout her subsequent writing. Her condition is always mentioned in the short biographical statement that all MOP respondents are encouraged to affix to the top of each directive response, allowing her to affirm her identity as a person living with a chronic illness through the very framing of her writing to an imagined reader. If Lucy is the perfect archetype of the active ‘service-user’ envisaged by current psychotherapeutic practice, Elizabeth, whose health fluctuated over the course of the 1980s and 1990s, can be read for examples of how MOP offers no satisfactory resolution. She finds writing about her experiences in the context of the 1997 NHS directive (DR 50) more comfortable, but also reveals that a second breakdown had occurred recently, as well as a longer-term experience of (previously unacknowledged) depression which runs through the chronology of her response to the 1992 ‘Pace of Life’ response. This may well indicate that her focus on being calm and healthy, with a consequent downplaying of her illness, in the earlier directive may well belie her attempt to achieve a true sense of composure.

The most challenging case of the value of reading MOP with attention to the non-linearity of memory comes in the case of Barbara. We have already noted her comparative descriptions of psychiatric hospitals at the end of the 1960s and the early 1980s. She remains reticent to discuss the cause of her hospitalisation in her NHS directive responses, but does address this in the 1994 ‘Drugs’ directive: a case of attempted rape by a man who spiked her drink with an unspecified drug. In her own conceptualisation, the trauma of assault was exacerbated by an adverse reaction to the drug, leading to her hospital stay.^134^ She acknowledged that she had never revealed to her parents that this had happened, and they consequently believed that her breakdown had occurred with no obvious trigger. Even her husband was only told the true cause in the same year as the directive, from which we might infer (although her description of disclosure in DR 43 is not clear on this) that her decision to recount this traumatic event for MOP allowed her sufficient narrative composure to reveal the truth to others.^135^ In the 2005 ‘Sex’ directive that so failed to elicit a response from Susan, Barbara was able to offer more detail about what had happened during her experience of sexual violence and shows that she had been able to construct a narrative which allowed her more control over the traumatic memory. She was able to reveal that the person who attempted to rape her was a fellow student and that, prior to this, she felt that the liberation of the ‘Sixties’ had ‘passed me by’. She then alluded to the effect of her nervous breakdown and that she ‘never returned to university because I just wasn’t the same person’, before using these experiences to pass judgement both on her attacker and male sexual violence generally: ‘What can be a “bit of fun” to a man can scar a woman for life and nearly destroy her mind and devastate her family. Maybe that man [i.e. her rapist] will think of all that if he has a daughter now himself’.^136^

All of these examples, to which many more might be added from the MOP archive, highlight the potential of researching MOP by reading several directives written by the same respondent (to obtain a narrative of an individual life) and then applying the tools of historical analysis. To the historical eye, a complex layering of different historical temporalities comes to bear: the linear narrative of an individual life and experience which can be marshalled together from evidence spread thickly and thinly through several directives; the more oscillating narrative of health, illness and affect which structures how an individual feels about their life at a given moment; and the narrative of memory which constructs and interprets events and feelings during and after their occurrence. Indeed, this latter point can itself be historicised, both by considering the moment of memory itself—the case of a writer in the 1990s recalling psychiatric institutionalisation in the 1960s, for example—and by interpolating our own position as historians. Barbara’s story takes on a new meaning to an audience post-2018, in which sexual violence has become more visible and open to challenge, and we are perhaps drawn to use her as an illustrative case precisely because she can address these issues. In Barbara’s case, something akin to closure and composure for her trauma came through her own identification (as even greater numbers of women would go on to do in the aftermath of ‘#MeToo’) as a survivor of violence who was not to blame. Of course, in writing about her memories in 1994 and 2005 she lacked the mass cultural script to enclose and consolidate this judgement.

Similarly, writing from the viewpoint of 2020 and a growing, and often unavoidable, publicization of mental health, it might be tempting to construct the entirety of our sample as voices adding to the ever-expanding confessional discourse,^137^ ‘outing’ themselves heroically (if perforce anonymously) as sufferers. This kind of present-centred thinking is highly problematic, but we invoke it here in order to draw attention to the possibility of MOP material to excavate a space for interrogating the complex layering and interplay of memory and lives. Involving the (researcher) reader in these chronologies opens up the nest of narratives within narratives that MOP so lucidly reveals. By providing a potential space for therapeutic acts and the assertion of individual agency, the MOP project allows individuals to construct self-narratives that may not, in some cases, have been shared with another human being and that can—sometimes with the help of shared cultural scripts—grant them forms of narrative closure.

## Conclusion and Discussion

This article has provided a brief outline of some of the voices and autobiographical records of experience that can be located within MOP and has proffered preliminary lines for how historians of mental health may wish to approach these longitudinally—both in terms of interpretation and practical methodology. By way of a conclusion, we shall attempt to extract three broad threads from our discussion of MOP lives, to show how the archive provides an important adjunct to our thinking about the history of mental health in post-war Britain.

First, MOP material allows us to begin to resituate the history of mental health as integral to life stories and biographies, rather than as something entirely framed by law, psychiatric medicine, and institutional provision.^138^ By considering mental health through narratives that oscillate between periods of ‘health’ and ‘illness’—and are, to some extent, structured *as narratives* by the interruptions and ‘disruptions’ that periods of severe illness exert on the writing process—the consequences of poor health can better be situated in the broader sequence of the life-course. One crucial aspect of sampling from MOP in relation to health—and mental health in particular—is that the individuals who come to the fore do not necessarily belong in neatly defined, and often externally imposed, categories. Even with oral history, the only other form of scholarly research which could elicit such personal testimony, participants are likely to have been sampled precisely because they fit an externally agreed and medically imposed label of ‘depression’, ‘anxiety’ or ‘stress’. Due to MOP’s open policy of accepting contributors to its mailing list without any pre-defined health characteristics—with only intermittent efforts at balancing the characteristics of potential respondents (much to the consternation of social scientists and more empirically minded historians)^139^—none of our sample have been pre-selected in this way. As a result of this, we witness a highly heterogenous set of individuals sometimes fighting against, sometimes militating in favour of, specific diagnostic categories (such as Lucy with her ME). What this illustrates, perhaps, is the fluidity between the nebulous categories of ‘health’ and ‘illness’. Whereas ‘mental illness’ is normally a category into which those diagnosed with certain conditions are forced by voices of expertise and authority, and into which they enter and leave—and only those within the category are likely to be chosen for research—MOP is as revealing about the other side of this: periods of health and functionality in between moments of anguish.

This can encompass Fiona’s efforts to seek employment-related retraining, Susan’s descriptions of raising her family, and the way in which these respondents live their lives against broader world events through the directives sent out by MOP to encourage writing about general elections or major national events. What matters here is that MOP’s more autobiographical mode of writing facilitates consideration of many different aspects of an individual, which ensures that health is always set within a rich context, both of personal living circumstances and wider social, political and economic situations. We created our sample through a set of directives on the NHS whose focus on biomedicine may well have primed respondents to construct and address their experiences through medicalised, psychological labels. This made locating such individuals a relatively straightforward process, since the nature of the directive favoured identification with wider popular labels of mental ill-health and therefore disclosure. We can nonetheless speculate about the future potential of employing the same methodology from different directive starting points within MOP to access less scientifically rationalised and more affective or emotions-based narratives of well-being.

Secondly, MOP’s longitudinal nature acts as a kind of psychodynamic window, allowing both the construction of narratives and self-reflection. The tension between ‘self’ and ‘experience ’as presented by a given respondent and any objective ‘reality’ is certainly marked in MOP, but it is useful to researchers because of this. It can be used to track changes over time and allows us to see, in accelerated motion, the influence of broader political junctures and cultural shifts on individuals’ framings of health and well-being, particularly by providing shared cultural scripts to validate memories and experiences. In doing so, it facilitates a mobilisation of age and generation alongside, and in productive dialogue with, the more common categories of identity. Although the linear process of ageing inevitably conditions responses to the various healthcare directives—as individual respondents draw on a memory-bank of past experiences—being healthy mentally and physically is as much constructed by memories of youth and maps onto changes in healthcare policy over the course of a lifetime. What some older generations of respondents experienced in terms of mental health care may be completely alien to those born just a few years later, when structural and institutional reforms had been brought to fruition. Indeed, our sample of respondents who explicitly mentioned mental illness cover a gamut of different treatment regimes, structured both by the years of their birth and the chronologies of their conditions: taking us from an era still dominated by psychiatric hospitals to the emergence of a series of new psychodynamic, behaviourist and psychopharmacological therapies, and the parallel struggle to access residential care. The value of the MOP material is that it demonstrates how individual biographies map unevenly and jarringly onto political and economic histories of mental health policy, medical histories of treatment and intellectual histories of psychiatric theory and practice. Once again, these are perforce underdeveloped in the spatial constraints of this article, but are certainly worthy of more sustained scholarly attention.

Thirdly, while stressing the need for historians to avoid retrospective diagnosis of lives in the past, MOP highlights the potential for the critical reading of responses for narrative closure and composure. This offers a way of assessing individual experiences of illness and therapy through the prism of memory, but also locating and contextualising them within the particular historical specificities of MOP itself. This allows us to maintain critical distance from a reified ‘experience’ whilst being aware of ‘voice’. Woods reminds us that ‘narratives’ must not be considered in isolation from their context as ‘transcultural, transhistorical’ modes of storytelling, nor as something open to everyone on the same terms.^140^ Only some of the MOP respondents were genuinely able to gain composure from their narratives; but they all did so within a very specific political and cultural context of life in Britain. This moment has witnessed an increasing individualisation of responsibility for mental health, and the rise of mental health campaigning (particularly around the turn of the Millennium). MOP may well feed into this moment, and the desires of individualisation which drive it; but it is equally a space for participants to provide personal reflection and offers access to a repository of autobiographical writing that can be read longitudinally (rather than as the product of a single juncture). This goes beyond the deep writings of Susan or Patricia and also engages the silences and refusals, of those who are more guarded (like many of the men in our sample), or those otherwise highly disclosing writers who were unable to engage with certain themes.

Lastly, however, we would like to stress the productive potential of the ‘accidental’ nature of the mental health material within the archive, arising from its primary function as a repository for heterogenous and mixed topics. Many historians have seen this as a challenge to accepted larger historiographical frames and theories. Hinton retreats from any attempt to impose a theoretical narrative on his M-O lives, instead proffering a ‘method’ (an ‘experiment in historiography’) based on weaving stories from the directive material around a degree of introspection about what attracts him to these lives.^141^ While in sympathy when faced with such rich autobiography material—and, at the same time, wishing to avoid succumbing to a psychologisation of our subjects rather than a historical analysis—we do wish to stress the ‘accidental’ nature of the archive as a point which may connect the wider theoretical concerns of the British mental health historiography. The latter is a diverse field of scholarship defined both by its concer with issues of psychiatric power and a critical engagement with Foucauldian framings of psychiatry through the act of nosological labelling^142^ as well as a longstanding social-historical alignment around axes of professionalisation, gender, class and race, which equally seeks to locate the patient as a vocalised agent.^143^ MOP provides one tool by which these traditions can be brought into dialogue; through focus on the construction of narrative over time and in relation to a variety of themes, and the intricate web of experience and power inhering around this.

In particular, employing MOP longitudinally may well offer a way of extending the work undertaken by Kirby, Haggett and Tracey Loughran, which position mental health alongside questions of gender and interpersonal relationships, as well as the broader currents of social and economic change which are dependent on such interconnected relations.^144^ Respondents may show evidence of absorbing particular models of discourses of mental health in given moments, but equally relate this to sometimes unexpected topics that set the researcher in a different direction. In forcing us to acknowledge our own positionality as researchers, MOP offers an opportunity to critically engage with an acknowledgement of these. While we have used individual MOP biographies, much as Hinton does, we have instead sought to bring these into dialogue with each other, rather than compartmentalising them. This, we suggest, is especially relevant to explore the commonalities of forming and writing about mental health. In conclusion, therefore, we urge historians of mental health in Britain—in all of its diversity—to pay attention to the longitudinal potential that inheres in MOP.

## Funding

This work was supported by the Wellcome Trust Senior Investigator Award held by Mathew Thomson and Roberta Bivins: ‘The Cultural History of the NHS’ [grant number: WT104837/Z/14/Z].

## Footnotes

1 Mass Observation Project Archive [hereafter MOA], Response to directive 15 (‘Social Well-Being’ [1984]), Susan.

2 See subsequent sections for our rationalisation of pseudonymising contributors.

3 Despo Kritsotaki, Vicky Long and Matthew Smith (eds), *Deinstitutionalisation and After: post-war psychiatry in the Western World* (Basingstoke: Palgrave Macmillan, 2016).

4 Joan W. Scott, ‘The evidence of experience’, *Critical Inquiry*, 1991, 17, 773–97. See also: Chris Millard, ‘Using Personal Experience in the Academic Medical Humanities: a Genealogy’, *Social Theory & Health*, 2020, 18, 184–98.

5 See: Nick Hubble, *Mass-Observation and Everyday Life: Culture, History, Theory* (Basingstoke: Palgrave Macmillan, 2006); Ben Highmore, *Ordinary Lives: Studies in the Everyday* (London: Routledge, 2011).

6 John Turner *et al.*, ‘The History of Mental Health Services in Modern England: Practitioner Memories and the Direction of Future Research’, *Medical History*, 2015, 59, 612–13.

7 Roy Porter, ‘The Patient’s View: Doing Medical History from Below’, *Theory and Society*, 1985, 14, 175–98; ibid., *A Social History of Madness: stories of the insane* (London: Phoenix Giants, 1999). For a critical reflection, see: Flurin Condrau, ‘The Patient’s View Meets the Clinical Gaze’, *Social History of Medicine*, 2007, 20, 525–40.

8 For a discussion of these themes, see: Emma L. Vickers, ‘Unexpected Trauma in Oral Interviewing’, *Oral History Review*, 2019, 46, 134–41.

9 Turner *et al.*, ‘The history of mental health services’, 602.

10 Angela Woods *et al.*, ‘The Recovery Narrative: Politics and Possibilities of a Genre’, *Culture, Medicine and Psychiatry*, 2019 [on-line only]; Angela Woods, ‘Rethinking “Patient Testimony” in the Medical Humanities: the Case of *Schizophrenia Bulletin*’s First Person Accounts’, *Journal of Literature and Science*, 2013, 6, 38–54; ibid., ‘The Limits of Narrative: Provocations for the Medical Humanities’, *Medical Humanities*, 2011, 37, 73–78; Claire Charlotte McKechnie, ‘Anxieties of Communication: The Limits of Narrative in the Medical Humanities’, *Medical Humanities*, 2014, 40, 119–24. For an outline of ‘narrative medicine’ by one of its exponents, see: Rita Charon, *Narrative Medicine: Honoring the Stories of Illness* (Oxford: OUP, 2006).

11 Grace K. Elliott *et al.*, ‘The Utilization of Cultural Movements to Overcome Stigma in Narrative of Postnatal Depression’, *Frontiers in Psychiatry*, October 2020; Elaine Hanzak, *Eyes Without Sparkle: A Journey through Postnatal Illness* (Abingson: Radcliffe, 2005); Carolyn Westall and Pranee Liamputtong, *Motherhood and Postnatal Depression: Narratives of Women and their Partners* (Dordrecht: Springer, 2011); Verta Taylor, *Rock-A-By Baby: Feminism, Self-help and Postpartum Depression* (London: Routledge, 1996). See also: Woods, ‘Rethinking “patient testimony”’ for how published narratives of schizophrenia are made to confirm and conform to medical authority.

12 Woods, et al., ‘The recovery narrative’, 5–6. For a feminist critique, see: Barbara Taylor, *The Last Asylum: a Memoir of Madness in our Times* (London: Hamish Hamilton, 2014); Joan Busfield, ‘Class and Gender in Twentieth-century British Psychiatry’, in Jonathan Andrews and Anne Digby (eds), *Sex and Seclusion, Class and Custody: Perspectives on Gender and Class in the History of British and Irish Psychiatry* (Amsterdam: Rodopi, 2004), 295–322.

13 In common with general usage, this article uses the hyphenated ‘Mass-Observation’ (M-O) to refer to the older of the two initiatives, and the unhyphenated Mass Observation Project (MOP) to refer to the new project from 1981 onwards. Various dates are proffered for the ‘end’ of the original M-O, which after 1948 mutated into a market research organisation.

14 Woods, ‘Rethinking “patient testimony”’, 40.

15 Woods *et al.*, ‘The recovery narrative’, 2–3, 13.

16 Penny Summerfield, ‘Culture and Composure: Creating Narratives of the Gendered Self in Oral History Interviews’, *Cultural and Social History*, 2004, 1, 65–93; Graham Dawson, *Soldier Heroes: British adventure, Empire and the imagining of masculinities* (London: Routledge, 1994).

17 Allan Ingram (ed.), *Voices of Madness: Four Pamphlets, 1683-1796* (Stroud: Sutton Publishing, 1997); Dale Peterson (ed.), *A Mad People’s History of Madness* (Pittsburgh: University of Pittsburgh Press, 1982). For a more nuanced account of women’s writing from the asylum see: Susannah Wilson, *Voices from the Asylum: Four French Women Writers, 1850-1920* (Oxford: OUP, 2010).

18 Jill Kirby, *Feeling the Strain: a Cultural History of Stress in Twentieth-century Britain* (Manchester: MUP, 2019).

19 Kirby, *Feeling the Strain*.

20 Tony Kushner, *We Europeans? Mass-Observation, “Race” and British Identity in the Twentieth Century* (Aldershot: Ashgate, 2004), 14–17.

21 Hinton, *Nine Wartime Lives*; Hinton, *Seven Lives from Mass Observation*; Kushner, *We Europeans?*; Andrea Salter, ‘Reading Time Backwards? Archival Research and Temporal Order’, in Niamh Moore *et al.*, *The Archive Project: Archival Research in the Social Sciences* (London: Routledge, 2017), 99–126. See also: Ken Plummer, *Documents of Life 2: An Invitation to Critical Humanism* (London: SAGE Publications, 2001).

22 Claire Langhamer, ‘Mass Observing the Atom Bomb: The Emotional Politics of August 1945’, *Contemporary British History*,2019, 208–25.

23 Lucy Noakes, ‘“My Husband is Interested in War Generally”: Gender, Family History and the Emotional Legacies of Total War’, *Women’s History Review*, 2018, 27, 610–626; ibid., ‘A Broken Silence? Mass Observation, Armistice Day and “Everyday Life” in Britain 19371941’, *Journal of European Studies*, 2015, 45, 331–46; Langhamer, ‘Mass observing the atom bomb’; Claire Langhamer, *The English in Love: The Intimate Story of an Emotional Revolution* (Oxford: OUP, 2013).

24 Kirby, *Feeling the Strain*, 8–9, 12–19.

25 Mike Savage and Magne Flemmen, ‘Life Narratives and Personal Identity: the End of Linear Social Mobility?’, *Cultural and Social History*, 2019, 16, 86–101.

26 Robert Dingwall, ‘Research Note: Thirty Years On’, *Sociology of Health and Illness*, 1979, 1:3, 348–50.

27 Helen Busby, ‘Writing about Health and Sickness: An Analysis of Contemporary Autobiographical Writing from the British Mass-Observation Archive’, *Sociological Research Online*, 2000, 5 ; Jenny Shaw, ‘Transference and Countertransference in the Mass-Observation Archive: An Under-exploited Research Resource’, *Human Relations*, 1994, 47, 1391–408.

28 Salter, ‘Reading time backwards?’, 110–14.

29 Summerfield, ‘Culture and composure’; Graham Dawson, *Soldier Heroes: British Adventure, Empire and the Imagining of Masculinity* (London: Routledge, 1994), 27–52; Shaw, ‘Transference and countertransference’.

30 Michael Bury, ‘Chronic Illness as Biographical Disruption’, *Sociology of Health and Illness*, 1982, 4, 167–82.

31 Ibid., 169.

32 Susan M. Benbow, ‘“Talking about my Experiences … at Times Disturbing yet Positive”: Producing Narratives with People Living with Dementia’, *Dementia*, 2016, 15, 1034–052; Edward Tolhurst *et al.*, ‘Narrative Collisions, Sociocultural Pressures and Dementia: The Relational Basis of Personhood Reconsidered’, *Sociology of Health and Illness*, 2017, 39, 212–26; Chris Sinding and Jennifer Wiernikowski, ‘Disruption Foreclosed: Older Women’s Cancer Narratives’, *Health: An Interdisciplinary Journal for the Social Study of Health, Illness and Medicine*, 2008, 12, 389–411; Annika Taghizadeh Larsson and Eva Jeppsson Grassman, ‘Bodily Changes among People Living with Physical Impairments and Chronic Illnesses: Biographical Disruption or Normal Illness?’, *Sociology of Health and Illness*, 2012, 34, 1156–69.

33 For a sociological critique of this aspect of Bury’s work, see: Larsson and Grassman, ‘Bodily changes’.

34 Sinding and Wiernikowski, ‘Disruption foreclosed’.

35 Katherine Foxhall, *Migraine: a history* (Baltimore: Johns Hopkins University Press, 2019), 14.

36 Emma Casey *et al.*, ‘Special Section Introduction: Mass Observation as Method’, *Sociological Research Online*, 2014, 19, para. 1.9; Rose Lindsey and Sarah Bulloch, ‘A Sociologist’s Field Notes to the Mass Observation Archive: A Consideration of the Challenges of “Re-using” Mass Observation Data in a Longitudinal Mixed-methods Study’, *Sociological Research Online*, 2014, 19. This is not without tensions. Lindsey and Bulloch note that historians and sociologists have frequently argued over Mass-Observation and sociologist’s ‘rendering of rich, emotive, individual “writing” or “material” into the “rational” “scientific” language of “data”’ (para. 4.1).

37 Mike Savage, ‘Changing Social Class Identities in Post-war Britain: Perspectives from Mass-Observation’, *Historical Social Research*, 2008, 33, 46–67; Florence Sutcliffe-Braithwaite, *Class, Politics, and the Decline of Deference in England, 1968-2000* (Oxford: OUP, 2018).

38 Savage and Flemmen, ‘Life narratives and personal identity’.

39 Dorothy Sheridan et al., *Writing Ourselves: Mass-Observation and literacy practices* (Cresskill: Hampton Press, 2000), 108, 116, 129–38.

40 Penny Summerfield, ‘Culture and composure’; Dorothy Sheridan, ‘Writing to the Archive: Mass-Observation as Autobiography’, *Sociology*, 1993, 27, 32.

41 Busby, ‘Writing about health and sickness’.

42 Ibid.

43 Though it is worthy of note that the archivists have seen an increase in those wishing to write for MOP post-COVID (personal communication from the archivist).

44 Lindsey and Bulloch, ‘A sociologist’s field notes’, para. 5.17.

45 Directive 112. Commissioned by the ‘Cultural History of the NHS’ project at the University of Warwick.

46 Directive 50 (‘You and the NHS’ [1997]) and Directive 82 (‘You & the NHS in 2008’).

47 The prompt read: ‘In recent years it has been argued that mental health services requires the same provision as physical illness services. Have you had any experiences in this area, and have you noticed changes over time? If so, in what way?’ (Directive 112). ‘Mental health’ was mentioned in 1997 and 2008, but not as a direct prompt: the former asked respondents to identify if ‘you or someone close to you … has a continuing need for health care for mental or physical illness’ (Directive 50), while the latter asked for respondents to describe ‘any specific health and social care experience’ from a list, which included ‘mental illness’ (Directive 82).

48 The total number of responses is 300, but one observer submitted two responses.

49 The archival boxes contain 250 responses, but there are two sets of duplicates.

50 These are the replies that have been processed and are now open to researchers.

51 Sheridan, et al., *Writing Ourselves*, 55-60.

52 Elliott, et al., ‘The utilization of cultural movements’; Hanzak, *Eyes Without Sparkle*. Indeed, some feminist scholars have argued that more women were drawn to see themselves through this label, leading to the illness becoming increasingly constructed socially: Paula Nicolson, *Post-Natal Depression: Psychology, Science and the Transition to Motherhood* (London: Routledge, 1998).

53 See: http://database.massobs.org.uk/projects_database/mass_observation/ (accessed on 25 April 2020).

54 These are available for free download at http://www.massobs.org.uk/mass-observation-project-directives (accessed 5 October 2020).

55 The directive, which was quite short, avoided ‘prompt[ing] you too much as part of the reason of asking you to do this is to see what you think ARE the key events’ of your life (Directive 82).

56 For an overview, see: Annebella Pollen, ‘Research methodology in Mass Observation Past and Present: “Scientifically, about as Valuable as a Chimpanzee’s Tea Party at the Zoo”?’, *History Workshop Journal*, 2013, 75, 213–35; Sheridan et al., *Writing Ourselves*, 55.

57 Sheridan et al., *Writing Ourselves*, 257–62.

58 Turner et al. identify this as a key departure point for writing new histories: ‘The history of the mental health services’, 612–13.

59 Busby, ‘Writing about health and sickness’.

60 Lucy employs this term, rather than the more common ‘chronic fatigue syndrome’; perhaps because its medical name suggests a more ‘scientific’ validation for the condition.

61 MOA, Response to Directive 81 (‘You and Gardens’ [2007]), Lucy.

62 MOA, Response to Directive 60 (‘Designing Your Own Directive’ [2000]), Lucy.

63 MOA, Response to Directive 82 (‘Your Life Line’ [2008]), Lucy.

64 MOA, Response to Directive 82 (‘You & the NHS in 2008’), Lucy.

65 MOA, Response to Directive 41 (‘Birth’ [1993]), Donna. For commonality of this narrative, see: Elliott, et al., ‘The utilization of cultural movements’, 10.

66 Ibid.

67 MOA, Response to Directive 50 (‘You and the NHS’ [1997]), Donna.

68 MOA, Response to Directive 60 (‘Designing Your Own Directive’ [2000]), Donna.

69 MOA, Response to Directive 36 (‘The Pace of Life’ [1992]), Fiona.

70 MOA, Response to Directive 43 (‘Drugs’ [1994]), Fiona.

71 MOA, Response to Directive 36 (‘The Pace of Life’ [1992]), Fiona.

72 MOA, Response to Directive 82 (‘You & the NHS in 2008’), Fiona. See also her response to DR 36 (‘The Pace of Life’ [1992]).

73 MOA, Response to Directive 36 (‘The Pace of Life’ [1992]), Fiona.

74 MOA, Response to Directive 50 (‘You and the NHS’ [1997]), Fiona.

75 MOA, Response to Directive 32 (‘Your Views on MO’ [1990]), Fiona.

76 MOA, Response to Directive 21 (‘AIDS Campaign’ [1987]), Fiona.

77 MOA, Response to Directive 23 (‘Holidays’ [1987]), Fiona.

78 Foxhall, *Migraine*, chapter 6, ‘“As sharp as if drawn with compasses”: Victorian vision, men of science, and the making of modern migraine’.

79 MOA, Response to Directive 82 (‘You & the NHS in 2008’), Fiona.

80 MOA, Response to Directive 90 (‘Childhood and Illness’ [2010]), Susan.

81 MOA, Response to Directive 103 (‘Dear 16 Year Old Me [2015]’), Susan.

82 MOA, Response to Directive 90 (‘Childhood and Illness’ [2010]), Susan.

83 MOA, Response to Directive 76 (‘Sex’ [2005]), Susan.

84 MOA, Response to Directive 82 (‘Your Life-Line’ [2008]), Susan.

85 MOA, Response to Directive 15 (‘Social Well-Being’ [1984]), Susan.

86 MOA, Response to Directive 41 (‘Birth’ [1993]), Susan.

87 MOA, Response to Directive 36 (‘Pace of Life’ [1992]), Susan.

88 MOA, Response to Directive 23 (‘Holidays’ [1987]), Susan.

89 MOA, Response to Directive 43 (‘Drugs’ [1994]), Susan.

90 MOA, Response to Directive 36 (‘The Pace of Life’ [1992]), Susan.

91 MOA, Response to Directive 43 (‘Drugs’ [1994]), Fiona.

92 Ibid.

93 Ibid.

94 MOA, Response to Directive 82 (‘Your Life-Line’ [2008]), Fiona.

95 MOA, Response to Directive 82 (‘You & the NHS in 2008’), Fiona.

96 Ibid., Trisha.

97 MOA, Response to Directive 43 (‘Drugs’ [1994]), Mike.

98 Ibid.

99 MOA, Response to Directive 82 (‘You & the NHS in 2008’), Jon.

100 Ali Haggett, *Desperate Housewives, Neuroses and the Domestic Environment, 1945-1970* (London: Pickering & Chatto, 2012); ibid., *A History of Male Psychological Disorders in Britain, 1945-1980* (Basingstoke: Palgrave Macmillan, 2015); Kirby, *Feeling the strain*. This is akin to how Hinton’s male subjects from the 1940s passed over emotionality in their wartime diaries: Hinton, *Nine Wartime Lives*, 151.

101 MOA, Response to Directive 82 (‘You & the NHS in 2008’), Mark.

102 Ibid.

103 MOA, Response to Directive 112 (‘You and the NHS’ [2018]), Robert.

104 MOA, Response to Directive 36 (‘The Pace of Life’ [1992]), Robert.

105 Mathew Thomson, *Psychological Subjects: Identity, Culture, and Health in Twentieth-Century Britain* (Oxford: OUP, 2006); Michal Shapira, *The War Inside: psychoanalysis, total war, and the making of the democratic self in postwar Britain* (Cambridge: CUP, 2013); Rhodri Hayward, *The Transformation of the Psyche in British Primary Care, 1870-1970* (London: Bloomsbury, 2015).

106 MOA, Response to Directive 49 (‘The Next General Election’ [1996/1997]), Elizabeth.

107 MOA, Response to Directive 36 (‘The Pace of Life’ [1992]), Elizabeth.

108 MOA, Response to Directive 50 (‘You and the NHS’ [1997]), Elizabeth.

109 MOA, Response to Directive 36 (‘The Pace of Life’ [1992]), Elizabeth.

110 MOA, Response to Directive 36 (‘The Pace of Life’ [1992]), Elizabeth.

111 Taylor, *The Last Asylum*.

112 MOA, Response to Directive 36 (‘The Pace of Life’ [1992]), Elizabeth.

113 MOA, Responses to Directive 50 (‘You and the NHS’ [1997]) and Directive 112 (‘You and the NHS’ [2018]), Patricia.

114 MOA, Responses to Directive 90 (‘Childhood and Illness’ [2010]) and Directive 103 (‘Dear 16 Year Old Me’ [2015]), Patricia.

115 MOA, Response to Directive 90 (‘Childhood and Illness’ [2010]), Patricia.

116 Ibid.

117 MOA, Responses to Directive 90 (‘Childhood and Illness’ [2010]) and Directive 76 (‘Sex’ [2005]), Patricia.

118 MOA, Response to Directive 103 (‘Dear 16 Year Old Me’ [2015]), Patricia.

119 MOA, Response to Directive 43 (‘Drugs’ [1994]), Barbara.

120 Ibid.

121 Busby, ‘Writing about health and sickness’, para. 9.

122 Sheridan et al., *Writing Ourselves*, 69–76.

123 Nick Hubble, *Mass-Observation and Everyday Life: Culture, History, Theory* (Basingstoke: Palgrave Macmillan, 2006), 14–15. This is also one reason why the original MO, unlike its present-day successor, is considered a ‘social movement’ by James Hinton: Hinton, *The Mass Observers: a history, 1937-1949* (Oxford: OUP, 2013).

124 Benbow and Kingston, ‘“Talking about my experiences…”’, 1047; Hubble and Tew; Louise Bazalgette et al., *Coming of Age* (London: Demos, 2011).

125 MOA, Response to Directive 32 (‘Your Views on MO’ [1990]), Susan.

126 MOA, Response to Directive 82, (‘Your Life-Line’ [2008]), Susan.

127 Sheridan et al., *Writing Ourselves*, 62–64.

128 MOA, Response to Directive 41, (‘Drugs’ [1994]), Susan.

129 MOA, Response to Directive 32 (‘Your Views on MO’ [1990]), Susan.

130 Ibid.

131 Salter, ‘Reading time backwards?’, 116–19.

132 MOA, Response to Directive 76 (‘Sex’ [2005]), Susan.

133 MOA, Response to Directive 41 (‘Birth’ [1993]), Fiona.

134 MOA, Response to Directive 43 (‘Drugs’ [1994]), Barbara.

135 Ibid.

136 MOA, Response to Directive 76 (‘Sex’ [2005]), Barbara.

137 For a discussion of this, see: Plummer, *Documents of Life 2*, 96–98.

138 The preparatory work for this article was slightly laborious in terms of recovering this, since it entailed multiple archival visits to MOP’s home at The Keep in Brighton, wading through a large quantity of material, and devising note-taking systems that could make this visible across multiple individuals. With the launch of a new digitised (and text-searchable) database for the 1980s (eventually to be expanded through to 2009) through MOP’s partnership with the publishers Adam Matthew Digital, however, part of this work can now be carried out remotely. See: ‘Adam Matthew Digital publishes the first module of Mass Observation Project, 1981-2009’, 8 July 2020. https://www.amdigital.co.uk/about/news/item/mass-observation-project-1981-2009 (accessed 5 October 2020).

139 We should note that policies on accepting new participants have evolved over time; with certain groups (middle-aged women living in southern England) actively discouraged at certain points. We are grateful to one of this article’s reviewers for raising the need for clarity on this quite complex point. Some background to this is given in Sheridan et al., *Writing Ourselves*, 58–60.

140 Woods, ‘The limits of narrative’, 74.

141 Hinton, *Nine Wartime Lives*, 1, 119–205.

142 Chris Millard, *A History of Self-Harm in Britain: A Genealogy of Cutting and Overdosing* (Basingstoke: Palgrave Macmillan, 2015); Rhodri Hayward, *Resisting History: Religious Transcendence and the Invention of the Unconscious* (Manchester: MUP, 2007); Colin Jones and Roy Porter (eds), *Reassessing Foucault: Power, Medicine and the Body* (Abingdon: Routledge, 1998); Mark Jackson, *The Age of Stress: Science and the Search for Stability* (Oxford: OUP, 2013). The earlier iterations of this trend was, of course, Nikolas Rose’s work, although Rose has since abandoned this in favour of more neurological and actor-network analysis: Nikolas Rose, *Governing the Soul: The Shaping of the Private Self*, 2nd edn (London: Free Association, 1999 [1989])

143 Roy Porter and Sarah Dunant (eds), *The Age of Anxiety* (London: Virago, 1996); Roy Porter and David Wright (eds), *The Confinement of the Insane: International Perspectives, 1800-1965* (Cambridge: CUP, 2003); Roy Porter, Helen Nicholson and Bridget Bennett (eds), *Women, Madness and Spiritualism* (Abingdon: Routledge, 2003); Porter, *A Social History of Madness*; Thomson, *Psychological Subjects*; Hilary Marland, *Dangerous Motherhood: Insanity and Childbirth in Victorian Britain* (Basingstoke: Palgrave Macmillan, 2004).

144 Kirby, *Feeling the Strain*; Haggett, *Desperate Housewives*; Tracey Lourghran and Dawn Mannay (eds), *Emotion and the Researcher: Sites, Subjectivities, and Relations* (London: Emerald Group Publishing, 2018).

